# Hyaluronic Acid-Based Gold Nanoparticles for the Topical Delivery of Therapeutics to the Retina and the Retinal Pigment Epithelium

**DOI:** 10.3390/polym13193324

**Published:** 2021-09-28

**Authors:** Amine Laradji, Bedia B. Karakocak, Alexander V. Kolesnikov, Vladimir J. Kefalov, Nathan Ravi

**Affiliations:** 1 Department of Ophthalmology and Visual Sciences, Washington University School of Medicine, St. Louis, MO 63110, USA; alaradji@wustl.edu (A.L.); karakocakb@wustl.edu (B.B.K.); 2 Department of Veterans Affairs, St. Louis Medical Center, St. Louis, MO 63106, USA; 3 Department of Ophthalmology, Gavin Herbert Eye Institute, University of California, Irvine, CA 92697, USA; sashakol@hs.uci.edu (A.V.K.); vkefalov@hs.uci.edu (V.J.K.); 4 Department of Physiology and Biophysics, University of California, Irvine, CA 92697, USA; 5 Department of Energy, Environmental and Chemical Engineering, Washington University in St. Louis, St. Louis, MO 63110, USA

**Keywords:** gold nanoparticles, hyaluronic acid, surface functionalization, transmission electron microscopy, inductively coupled plasma mass spectrometry, biocompatibility, ocular drug delivery, retina, retinal pigment epithelium, retinal diseases

## Abstract

The ocular immune privilege is a phenomenon brought about by anatomical and physiological barriers to shield the eye from immune and inflammation responses. While this phenomenon is beneficial for eyes protection, it is, at the same time, a hindrance for drug delivery to the posterior segment of the eye to treat retinal diseases. Some ocular barriers can be bypassed by intravitreal injections, but these are associated with several side effects and patient noncompliance, especially when frequent injections are required. As an alternative, applying drugs as an eye drop is preferred due to the safety and ease. This study investigated the possible use of topically-applied hyaluronic acid-coated gold nanoparticles as drug delivery vehicles to the back of the eye. The coated gold nanoparticles were topically applied to mouse eyes, and results were compared to topically applied uncoated gold nanoparticles and phosphate-buffered saline (PBS) solution. Retina sections from these mice were then analyzed using fluorescence microscopy, inductively coupled plasma mass spectrometry (ICP-MS), and transmission electron microscopy (TEM). All characterization techniques used in this study suggest that hyaluronic acid-coated gold nanoparticles have higher distribution in the posterior segment of the eye than uncoated gold nanoparticles. Electroretinogram (ERG) analysis revealed that the visual function of mice receiving the coated gold nanoparticles was not affected, and these nanoparticles can, therefore, be applied safely. Together, our results suggest that hyaluronic acid-coated gold nanoparticles constitute potential drug delivery vehicles to the retina when applied noninvasively as an eye drop.

## 1. Introduction

The posterior segment of the eye comprises the sclera, the retina, the choroid, the optic nerve, and the vitreous humor. This part of the eye is susceptible to several disorders and diseases that often lead to irritation, visual impairment, and even vision loss, such as glaucoma, age-related macular degeneration, and retinopathy [1]. Low bioavailability from systemic administration has made delivering drugs to the retina challenging due to various ocular barriers [2,3,4,5], hindering penetration to the posterior segment of the eye. The drug transport through ocular layers is anatomically hindered by the corneal, conjunctival, and epithelial cells forming tight junctions [6]. On the other side, drugs applied on the eye surface are prone to dilution and removal by tear, lacrimation, and blood flow, decreasing the drug’s residence time and absorption on the eye surface. Successful topical delivery of specific small molecules has been observed [7,8]. However, the instillation of much larger molecules is problematic because of the decreasing permeability with increasing molecular weight and size [9].

In clinical practices, intravitreal injection is the current mode of delivery to the retina. This procedure is accompanied by various side effects such as inflammation, retinal damage, and poor localization [10,11,12,13]. Therefore, developing novel targeted drug delivery vehicles is highly desired. The over-expression of a cluster of differentiation 44 (CD44) receptors is a common feature of many ocular diseases [14,15,16]. Retinal pigmented epithelium (RPE) [17] retinae [16], Müller cells [18], and ganglion cells express CD44 receptors in their normal state and over-express them in disease conditions. CD44 receptors have an affinity for hyaluronic acid (HA) that enables cells to adhere and anchor themselves or to internalize HA [19,20,21,22], depending on its molecular weight [23]. Thus, coating nanoparticles with HA would deliver more drugs to cells that over-express CD44 receptors and enable Receptor-Mediated Endocytosis (RME), thereby providing statistical specificity. Although any drug-nanoparticle conjugates can be coated with HA, here we provide proof of concept using gold nanoparticles (AuNPs) because their size, shape, and surface properties can be precisely altered. Their unique surface plasmon resonance (SPR) effect can also be used for imaging [24], photothermal therapy [25], and anti-angiogenic therapeutic applications [26]. During choroidal neovascularization (CNV), endothelial cells over-express CD44 and vascular endothelial growth factor (VEGF), and therefore, the innate anti-angiogenic activity of AuNPs can be tested [27]. Recently, the anti-angiogenic properties of gold nanoparticles were compared to HA-coated gold nanoparticles for the treatment of intraocular vascular disorders via intraretinal injection [27]. Interestingly, the anti-angiogenic properties of the two populations were not significantly different. However, HA-coated gold nanoparticles showed better diffusion through the posterior eye. They could reach deeper retinal layers than uncoated gold nanoparticles, making them potentially good candidates for carrying therapeutics to the retina. Nevertheless, these nanoparticles were delivered via an intravitreal injection, which carries the risk of post-injection complications associated with similar drug delivery means.

Topically applied small molecules may cross ocular barriers to reach the retina. However, the topical application of large macromolecules, such as CRISPR-cas9 and siRNA, has not been successful in delivering therapeutics to the retina as these large molecules are blocked from passive cell internalization and passage through all of the natural ocular barriers [28].

Our research group has recently demonstrated that the biocompatibility of gold nanoparticles and their cell internalization originate from an interaction between the nanoparticles size and surface chemistry [29]. Coating gold nanoparticles with hyaluronic acid (HA) significantly increases the biocompatibility of nanoparticles and enables larger nanoparticles (i.e., carrying larger cargo) to enter the cells. A key novel concept is that our method relies on the affinity of hyaluronic acid for the cell-surface receptor CD44. These receptors internalize HA and any particles coated with HA. While this concept is being developed in cancer therapy [30], its application in ophthalmology is novel and remains unexplored.

In the present work, we investigated the use of hyaluronic acid-coated gold nanoparticles as potential vehicles for delivering therapeutics to the posterior segment of the eye via an eye drop. The gold nanoparticles were prepared according to our previously reported protocol [29], and had an average diameter of 15–20 nm. Hyaluronic acid was grafted on the surface of the nanoparticles via a gold-thiol reaction [31]. Taking advantage of their surface plasmon resonance, the distribution of HA-AuNPs throughout ocular layers was examined using fluorescence microscopy. To confirm that HA-AuNPs reached the posterior part of the eye, the delivered nanoparticles were analyzed with inductively coupled plasma mass spectrometry (ICP-MS) and observed with transmission electron microscopy (TEM). In these experiments, uncoated AuNPs were used as a control to highlight the effects of coating the nanoparticles with HA.

## 2. Materials and Methods

### 2.1. Animals

Wild-type mice with a 129S2/Sv genetic background were obtained from Charles River Laboratories. Adult animals of either sex (4-month-old) were used. Animals were provided with standard chow (LabDiet 5053; LabDiet, Purina Mills, St. Louis, MO, USA) and maintained under a 12 h light/12 h dark cycle. Mice were dark-adapted overnight before the physiological recordings. All experimental protocols followed the Guide for the Care and Use of Laboratory Animals and were approved by the Washington University Animal Studies Committee (Protocol No 20-0181, approval date: 1 March 2021).

### 2.2. Synthesis and Preparation of the Gold Nanoparticles and Their Coating with HA

All experimental work that includes synthesizing gold nanoparticles, their layer with HA, and their characterization is provided in our previously published article [29]. Briefly, 10 mg of gold (III) chloride hydrate (HAuCl_4_) was dispersed in 90 mL of deionized water, followed by boiling the solution. Sodium citrate solution (250 mM) was added, followed by stirring for 20–30 min, after which the solution became wine-red in color. This solution was then left in the dark for 24 h at room temperature with no stirring. Finally, the nanoparticles solution was centrifuged at 10,000 rpm for 20 min and redispersed in DI water. In the next step, thiol-modified HA chains were conjugated to the surface of the gold nanoparticles by adding them to the Au-NPs solution at room temperature with moderate stirring. After overnight stirring, the HA-modified gold nanoparticles solution was purified via centrifugation at 10,000 rpm for 20 min. The modified gold nanoparticles were, finally, redispersed in DI water.

### 2.3. Confocal Microscopy for Detection of Au NPs

First, 3 µL of HA-coated or uncoated AuNPs (0.05 mg/mL) were applied topically on mouse eyes. Thirty minutes later, the mice were sacrificed by CO_2_ asphyxiation, and eyes were removed. The extracted eyes were fixed in a solution of 4% paraformaldehyde overnight, then processed through graded alcohol and cleared with xylenes, and infiltrated through four changes of paraffin. The eyes were embedded in a sagittal manner. The sections were cut at 4 μm thickness on a rotary microtome, and slides were dried for 30 min at 60 °C. Finally, the slides were visualized under a confocal microscope Zeiss LSM800 (Carl Zeiss Microscopy, Jena, Germany) using a 488 nm excitation wavelength and 500–550 nm emission wavelength to investigate AuNPs distribution. The slides did not go through any staining procedure. For Au NPs in the size range of 5–100 nm, the SPR wavelength is around 520 nm [32], depending on the sizes of the nanoparticles. See the supporting information for the details.

### 2.4. Inductively Coupled Plasma Analysis for the Confirmation of Gold Nanoparticles Presence

Three µL of HA-AuNPs was applied topically on the right eye of the mouse, and the same amount of AuNPs was applied to the left eye. Thirty minutes after application, the animals were sacrificed and their eyes extracted. After removing the vitreous, the eyes were crosslinked in a solution of 4% paraformaldehyde overnight, followed by their complete digestion in aqua regia. Next, the solution was completely evaporated, and HNO_3_ (1% solution in DI water) was added. Next, this solution was filtered through a 0.45 µm filter, then injected into ICP-MS (NexION 2000, PerkinElmer, Inc., Waltham, MA, USA) for its analysis. The amount of gold was determined from a calibration curve plotted after injection of several concentrations of a gold standard solution.

### 2.5. Transmission Electron Microscopy Imaging for Visualization of Gold Nanoparticles’ Distribution throughout the Retina

Eye tissue was fixed in a modified Karnovsky’s fixative of 3% glutaraldehyde, 1% paraformaldehyde in 0.1 M sodium cacodylate buffer. The tissue was then post-fixed in 2% osmium tetroxide in 0.1 M sodium cacodylate buffer for 1 h, en bloc stained with 3% aqueous uranyl acetate for 30 min, dehydrated in graded ethanol, and embedded in PolyBed 812 catalog # 08792-1 (Polysciences, Hatfield, PA, USA).

Tissue blocks were sectioned at 90 nm, immediately placed on a 400 mesh carbon film-supported copper TEM grid (Sigma-Aldrich cat# TEM-CF400CU50, St. Louis, MO, USA), post-stained with Venable’s lead citrate, and viewed with a JEOL model 1400EX electron microscope (JEOL, Tokyo, Japan). Digital images were acquired using the AMT NanoSprint 12A-B (Advanced Microscopy Technology, Danvers, MA, USA) CMOS, 12 megapixel TEM camera.

### 2.6. In Vivo ERG Analysis after Topical Application of Gold Nanoparticles

Dark-adapted wild-type mice were anesthetized with an intraperitoneal injection of a mixture of ketamine (100 mg/kg) and xylazine (20 mg/kg). Three µL of HA-AuNPs was applied topically to the right eye of each mouse placed on a heating pad of the ERG apparatus to maintain its body temperature at 37 °C. Control left eyes were treated with an equivalent volume of PBS. After 30 min, the anesthesia was repeated with ~1/2 of the initial dose of ketamine. Ten µL of PBS was then applied to each eye to maintain the cornea wet. Measurements were started after an additional 30 min (for a total time of 1 h after the beginning of topical applications). Pupils were dilated with a drop of 1% atropine sulfate. ERG responses were recorded from both eyes by corneal contact electrodes held in place by a drop of Gonak solution. Full-field ERGs were performed with the UTAS BigShot apparatus (LKC Technologies, Gaithersburg, MD, USA) using Ganzfeld-derived test flashes of calibrated green 530 nm LED light (within a range from 2.5 × 10^−5^ cd∙s m^−2^ to 23.5 cd∙s m^−2^) or white light generated by the Xenon Flash tube (from 80.7 cd∙s m^−2^ to 700 cd∙s m^−2^). Both ERG a-waves and b-waves were evaluated. Data were expressed as means ± SEM and analyzed using the independent two-tailed Student’s t-test, with an accepted significance level of *p* < 0.05.

## 3. Results and Discussion

Gold nanoparticles were identified as a potential drug delivery system for this study. Drugs can be loaded on gold nanoparticles either physically or chemically. One example of physical loading is the electrostatic interaction between a positively charged drug and a negatively charged polymer, such as hyaluronic acid, tethered to the surface of the AuNPs. Chemically, drugs can also be attached to gold nanoparticles covalently either directly on their surface using a thiol-Au bond or by conjugating drugs to a polymer already attached to AuNPs. This approach requires that the drug and the polymer have complementary functional groups for a covalent attachment. As stated above, AuNPs can easily be altered to obtain desired physical and chemical properties. They mainly possess the surface plasmon resonance (SPR) effect used in this study for imaging [24]. Because of the strong gold-thiol interaction, the coating on the gold nanoparticles is stable [31]. This bond is in the order of a covalent bond. Over time, however, this coating may degrade due to the inherent susceptibility of hyaluronic acid to degrade through oxidative damage. Before that, however, HA was end-modified by cystamine, followed by cystamine reduction to make end-thiolated hyaluronic acid chains. HA is a natural anionic electrolyte and the primary ligand for cell receptors CD44 [33,34]. Because of its affinity for HA, CD44 enables cells to adhere to HA in a ground substance. This particular affinity has been taken to design biomaterials for several medical applications such as bioimaging and drug delivery [35,36]. Other benefits of using HA include its biocompatibility and good aqueous solubility [37,38]. Finally, HA has carboxyl groups in its repeat units, which can attach therapeutic agents to its backbone either via covalent bonds or electrostatic interactions.

**Figure 1:**
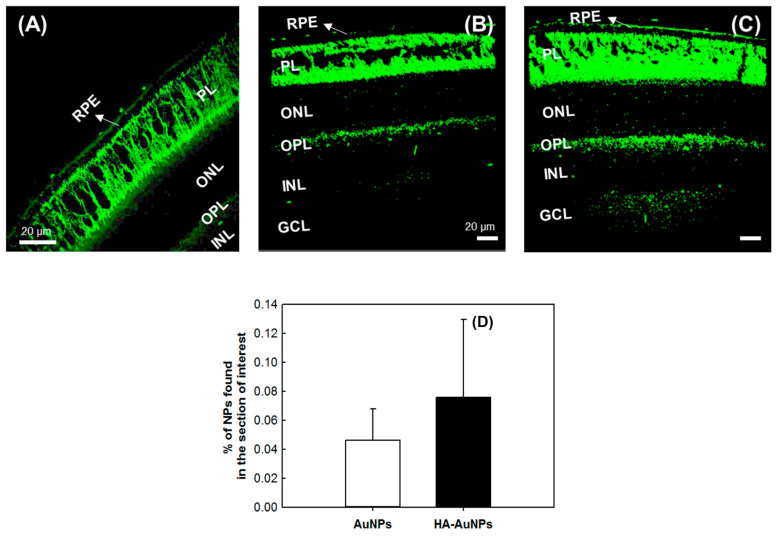
Confocal microscopy images of retinal sections from non-treated mice as a control sample (**A**), animals treated with AuNPs (**B**), and HA-AuNPs (**C**). Resultingly, ~50% more HA-AuNPs were detected in the retina within 5 min (**D**). (*n* = 3). RPE: retinal pigment epithelium, PL: photoreceptors layer, ONL: outer nuclear layer, OPL: outer plexiform layer, INL: inner nuclear layer, GCL: ganglion cell layer.

To determine whether coating AuNPs with HA improves their delivery to the posterior side of the eye, a solution of 0.05 mg/mL of HA-AuNPs in PBS was applied topically on the right eyes of mice. In contrast, the left eyes received uncoated AuNPs as a control. As seen in Figure 1, confocal microscopy imaging revealed an increase in fluorescence signal that reflects the successful delivery of HA-AuNPs into the retinal layers and, more specifically, the RPE layer. We estimated that 50% more of HA-AuNPs were detected in the retina than the control sample.

Cellular internalization and biodistribution of gold nanoparticles are dependent on their size. Kim and colleagues showed that uncoated gold nanoparticles with 20 nm can cross the blood-retinal barrier and reach the retina layers when injected intravenously [39]. Gold nanoparticles with 100 nm were not detected in the retina [40,41]. This trend, however, can be affected by the surface chemistry of the NPs. Our previous study observed in vitro that coating AuNPs with HA enables larger NPs to enter the cells, which they otherwise could not do [29]. More specifically, we demonstrated that HA coating facilitates spherical AuNPs that possess diameters higher than 50 nm into ARPE-19 cells, which express CD44 receptors during their proliferation. Consistently with that, Apaolaza et al. observed that the mobility of gold nanoparticles through the vitreous humor was improved upon their coating with HA, which resulted in an increased distribution of the NPs throughout the retinal layers [27]. This observation can be ascribed to several factors, including the size of AuNPs, and the negative charge and hydrophilicity of HA. These physical characteristics likely enabled AuNPs to traverse through the scleral layer, which is porous, hydrophilic, and negatively charged [5]. On the other side, CD44 cell receptors expressed in the RPE layer allowed the AuNPs to target these cells via cell receptor-mediated endocytosis.

**Figure 2:**
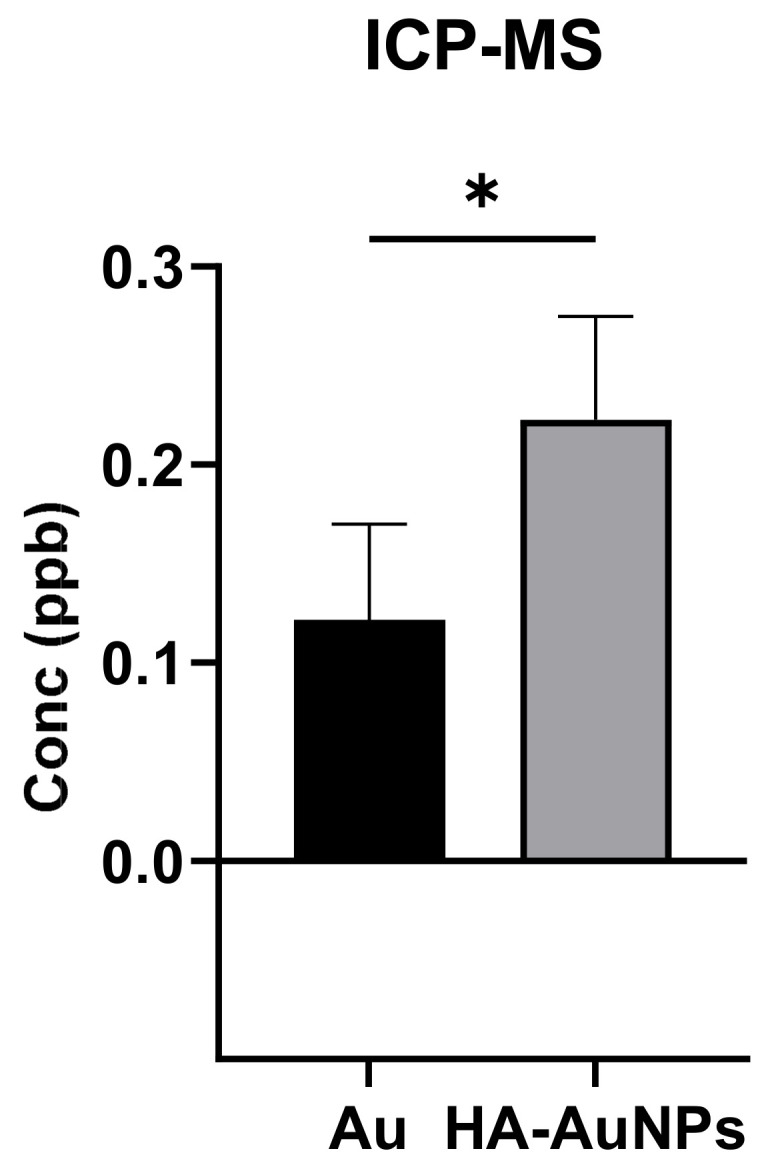
Analysis of the posterior part of the eye by ICP-MS showing that HA coating indeed improves NPs distribution. Statistical significance of this date is presented as * *p* < 0.05.

To confirm the effect of HA on the delivery of AuNPs to the retina, ICP-MS was used to detect gold traces in the back of the eye after topically applying a solution of the NPs. We used ICP-MS to estimate the amount of coated and uncoated gold nanoparticles that reached the back of the eye. Thirty minutes after topically applying the NPs, mouse eyes were extracted, and their vitreous was removed. The remaining tissues were analyzed after their complete digestion in aqua regia. As demonstrated in Figure 2, the amount of HA-AuNPs in the tissue was significantly higher than that of uncoated AuNPs, corroborating our results obtained from confocal microscopy.

**Figure 3:**
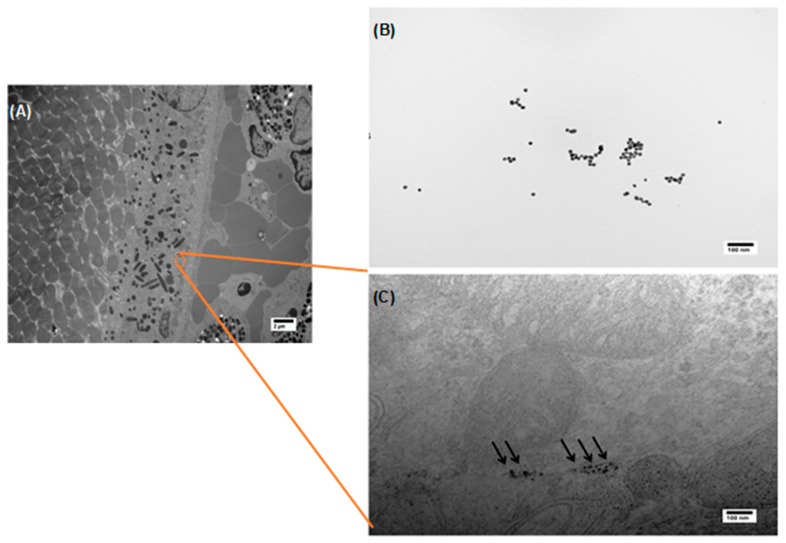
TEM images showing HA-AuNPs biodistributed in the RPE layer, 30 min following their instillation on the surface of a mouse eye. (**A**) Micrograph of a cross-section through the choroid, RPE, and the retina of mouse treated with HA-AuNPs, (**B**) HA-AuNPs, and (**C**) HA-AuNPs within the RPE layer.

TEM was used to confirm the presence of HA-AuNPs, more specifically in the RPE layer. This electron microscopy technique is a powerful imaging tool to visualize the biodistribution of nanoparticles and was previously used to image the layers of the retina for different animals [42,43,44]. Indeed, in Figure 3, the NPs can be clearly distributed in the RPE layer, further confirming their ability to cross the various anatomical and physiological barriers. The RPE layer is a cellular monolayer that is strategically located between the choroid and the retinal photoreceptors. This cellular layer, which plays critical roles that involve the transport of nutrients and maintaining the retinoid (visual) cycle, can also cause several ocular diseases such as Leber congenital amaurosis type 2 that originates from various RPE65 mutations. Therefore, targeting the RPE layer successfully for drug delivery purposes is critical for restoring visual function [45,46]. Clinically, intravitreal injection is commonly used to deliver therapeutics to the posterior eye. However, this method is accompanied by side effects exacerbated when these injections are frequently administered.

Therefore, targeting the RPE layer using topically applied formulations is safer and would improve patient compliance. It is worth noting that TEM imaging did not reveal any structural anomaly of cells that were in contact with the nanoparticles, which is in concordance with our previous findings where we observed that HA coating significantly enhances the viability of human ARPE-19 cells in vitro upon their exposure to HA-AuNPs [29].

**Figure 4:**
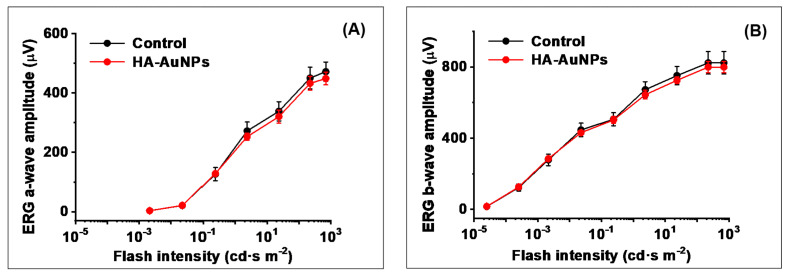
Topical application of HA-AuNPs does not affect scotopic ERG a-wave (**A**) and b-wave (**B**) responses of mice in vivo. Averaged rod intensity-response functions (mean ± SEM, *p* > 0.05 for all data points) for control PBD-treated wild-type mouse left eyes (*n* = 4) and right eyes of the same animals treated with HA-AuNPs (*n* = 4). Recordings were performed 1 h after treatment.

Finally, we investigated, by ERG, whether HA-AuNPs delivery has any detrimental effect on the visual function of the retina in vivo. In this experiment, four wild-type mice received HA-AuNPs topically on their right eyes, while the control left eyes were administered with phosphate-buffered saline (PBS) solution. Importantly, we found no reduction of both scotopic ERG a-wave (initial negative component driven by rod photoreceptors) and b-wave (larger positive ERG component driven by rod ON bipolar cells) responses at any tested light intensity, for up to 1 h after the HA-AuNPs application (Figure 4). The rod sensitivity of NP-treated mice also remained unchanged. These results clearly indicate the safety of HA-AuNPs treatment for the overall visual function in mice.

## 4. Conclusions

In conclusion, this communication reports the successful delivery of hyaluronic acid-coated gold nanoparticles to the retina via topical application. Fluorescence microscopy, ICP-MS, and transmission electron microscopy revealed that the applied nanoparticles can effectively reach the retina layers of the eye and remain there with no adverse effects on cells structure and retinal function, as confirmed by ERG recordings. The use of hyaluronic acid-coated gold nanoparticles as a drug delivery system constitutes a promising alternative to the commonly used route for administering various therapeutics to the posterior segment of the eye. The HA-coated nanoparticles reported here display desirable properties of drug delivery vehicles such as non-toxicity, good biodistribution, and active targeting of required sites through hyaluronic acid-CD44 affinity. These desired properties will make them excellent candidates to deliver drugs to the posterior eye segment, and more specifically, to the RPE cells and the retina.

## Data Availability

All data is available within the manuscript.
